# A dual diversity perspective on individual response variability in tDCS motor learning research: Some comments on Blake et al., 2026

**DOI:** 10.14814/phy2.71162

**Published:** 2026-10-28

**Authors:** Thorsten Rudroff

**Affiliations:** ^1^ Turku PET Centre University of Turku and Turku University Hospital Turku Finland

Blake and colleagues are to be commended for their transparent reporting of a null finding in a well‐designed study examining whether anodal transcranial direct current stimulation (a‐tDCS) over primary motor cortex (M1) enhances acquisition of a dexterous, timing‐based videogame task (Blake et al., 2026). Their honest acknowledgment that individual response variability made group‐level interpretation “nearly impossible” is scientifically valuable—but it also points to an unresolved methodological problem that deserves explicit attention and that, I argue, may reframe the null finding itself.

## 1 THE GROUP MEAN AS A METHODOLOGICAL ARTIFACT

The randomized, sham‐controlled, single‐blind design employed by Blake et al. represents current best practice in tDCS motor learning research. Both groups improved significantly across all three performance measures: accuracy, best continuous streak, and overstrums, with large effect sizes (*ηp* ^2^ = 0.451 to 0.801), confirming robust motor skill acquisition with practice. Yet no group differences emerged at any time point, and Bayes factors ranging from 0.66 to 0.98 provided only weak evidence against a true effect. The authors invoke individual neuroanatomical variability, montage‐specific current flow, and baseline excitability state as likely moderators. This is a reasonable interpretation, but one that stops short of identifying the biological sources of that variability.

This interpretive gap reflects a broader structural problem in motor learning and neuromodulation research. When samples are drawn predominantly from young, healthy, university‐affiliated adults and analyzed as undifferentiated groups, biologically meaningful individual differences are averaged out of existence. The result is not a clean null finding but a statistically indeterminate one, precisely what the authors' TOST equivalence analysis confirmed, with a 90% confidence interval spanning from −21.07 to 19.47, far exceeding the predefined equivalence boundaries. This is not evidence that a‐tDCS does not work; it is evidence that the current analytical framework was insufficiently sensitive to detect structured heterogeneity in treatment response.

## 2 THE DUAL DIVERSITY DEFICIT

I have previously described this problem as the Dual Diversity deficit, encompassing two distinct but interacting dimensions: population‐level demographic homogeneity, in which research samples systematically exclude older adults, females, and individuals with neurological conditions; and individual neurobiological heterogeneity, in which genuine biological differences among participants are treated as noise rather than signal (Rudroff, 2026a, 2026b). Both dimensions are present in the current study. The sample of forty healthy young adults with a mean age of 22 years, recruited from a single university setting, is demographically narrow by design, consistent with standard practice but structurally limited in its generalizability. More critically, biological sex is neither reported as a stratifying variable in the results nor discussed as a potential moderator of tDCS response, despite substantial evidence that sex systematically structures cortical excitability, motor cortex organization, and neuromodulatory responsiveness (Pitcher et al., 2003; Rivas‐Grajales et al., 2023; Rudroff et al., 2020).

## 3 BIOLOGICAL SEX AS A STRUCTURED MODERATOR

The omission of biological sex as an analytical variable is particularly consequential in the context of tDCS research. Sex differences in corticospinal physiology are documented but heterogeneous across measures. Some TMS studies report no consistent sex difference in group‐level motor‐evoked potential amplitude, yet females show greater trial‐to‐trial response variability than males (Pitcher et al., 2003), and males show greater intracortical inhibition than females on some paired‐pulse measures even when excitability itself does not differ (Kells et al., 2026), indicating that sex structures specific components of corticospinal physiology rather than acting as a uniform effect. Critically, hormonal fluctuations across the menstrual cycle further modulate cortical excitability and tDCS response in ways that are rarely controlled for in motor learning studies (Rivas‐Grajales et al., 2023; Rudroff et al., 2020; Smith et al., 1999). I have previously demonstrated that high estrogen levels are associated with significantly greater leg muscle fatigability following 4 mA tDCS in eumenorrheic young women, directly implicating sex hormone status as a moderator of neuromodulatory response magnitude (Deters et al., 2022). These are not minor sources of variance; they are systematic biological signals that, when unexamined, inflate within‐group variability and reduce the sensitivity of between‐group comparisons.

In my own FDG‐PET neuroimaging work examining motor‐relevant subcortical circuitry, biological sex accounted for 29.71% of globus pallidus metabolic variance (*η* ^2^ = 0.2971; F (1,30) = 12.68; *p* = 0.0013; *d* = 1.26) while fatigue status explained essentially none (Rudroff, 2026b). The globus pallidus is a key node in the cortico‐striatal‐thalamo‐cortical loops that support motor sequence learning and consolidation, the very circuits implicated in the task demands of the Guitar Hero paradigm. If biological sex structures metabolic activity in this circuitry to this degree, it is reasonable to hypothesize that it also structures individual responsiveness to M1 a‐tDCS during dexterous motor learning.

## 4 A CONSTRUCTIVE RECOMMENDATION

I respectfully suggest that a re‐analysis of the existing dataset stratified by biological sex may reveal differential tDCS response patterns that the current group‐level design was structurally unable to detect. The study enrolled 22 male and 18 female participants—sufficient for an exploratory sex‐stratified analysis, with appropriate caution regarding statistical power. If such differences exist, the null finding would be reframed not as evidence against M1 a‐tDCS efficacy, but as evidence that efficacy is individual‐specific and biologically structured. If no differences emerge after stratification, that negative result would itself be informative and publishable, strengthening the authors' original conclusion on firmer analytical ground.

More broadly, I encourage the field to move beyond the treatment of individual variability as a limitation to be acknowledged in a final paragraph and toward its explicit characterization as a primary research question (Rudroff, 2026a). The substantial individual scatter visible in Figures 3 through 5 of the Blake et al. paper is not methodological noise; it is a dataset waiting to be analyzed. Identifying who responds to M1 a‐tDCS, under what biological conditions, and through which neural mechanisms is a more tractable and ultimately more clinically relevant question than whether a‐tDCS works on average across a homogeneous young adult sample.

The Guitar Hero paradigm developed by Blake and colleagues is innovative, reproducible, and well‐suited to quantifying dexterous motor learning. Embedding individual neurobiological heterogeneity as an explicit variable, rather than a limitation, would substantially increase its scientific yield and its relevance to populations beyond the young, healthy, university‐affiliated adult.

## AUTHOR CONTRIBUTIONS

**Thorsten Rudroff:** Conceptualization.

## FUNDING INFORMATION

The author declares no funding for this work.

## CONFLICT OF INTEREST STATEMENT

The author declares no competing interests.

## ETHICS STATEMENT

None.
